# Urinary metabolic profile and its predictive indexes after MSG consumption in rat

**DOI:** 10.1371/journal.pone.0309728

**Published:** 2024-09-03

**Authors:** Manatsaphon Sukmak, Thin Su Kyaw, Kanokwan Nahok, Amod Sharma, Atit Silsirivanit, Worachart Lert-itthiporn, Deanpen Japrung, Somchai Pinlaor, Sirirat Anutrakulchai, Carlo Selmi, Carolyn M. Slupsky, Bruce D. Hammock, Ubon Cha’on

**Affiliations:** 1 Department of Biochemistry, Faculty of Medicine, Khon Kaen University, Khon Kaen, Thailand; 2 Chronic Kidney Disease prevention in the Northeast of Thailand (CKDNET), Khon Kaen University, Khon Kaen, Thailand; 3 Department of Pharmacology and Toxicology, College of Medicine, University of Arkansas for Medical Sciences, Little Rock, AR, United States of America; 4 National Nanotechnology Center (NANOTEC), National Science and Technology Development Agency (NSTDA), Thailand Science Park, Pathum Thani, Thailand; 5 Department of Parasitology, Faculty of Medicine, Khon Kaen University, Khon Kaen, Thailand; 6 Department of Internal Medicine, Faculty of Medicine, Khon Kaen University, Khon Kaen, Thailand; 7 Rheumatology and Clinical Immunology, IRCCS Humanitas Research Hospital, Rozzano, Milan, Italy; 8 Department of Biomedical Sciences, Humanitas University, Pieve Emanuele, Milan, Italy; 9 Department of Nutrition and Department of Food Science & Technology, University of California, Davis, CA, United States of America; 10 Department of Entomology & Nematology and the UC Davis Comprehensive Cancer Research Center, University of California, Davis, CA, United States of America; NED University of Engineering and Technology, PAKISTAN

## Abstract

Monosodium glutamate (MSG) is a widely used food additive with conflicting evidence regarding its potential effects on human health, with proposed relevance for obesity and metabolic syndrome (MetS) or chronic kidney disease. As being able to accurately quantify the MSG dietary intake would help clarify the open issues, we constructed a predictive formula to estimate the daily intake of MSG in a rat model based on the urinary metabolic profile. Adult male Wistar rats were divided into groups receiving different daily amounts of MSG in drinking water (0.5, 1.5, and 3.0 g%), no MSG, and MSG withdrawal after 3.0% MSG treatment for 4 weeks. We then analyzed 24-hour urine samples for chemistries and metabolites using ^1^H NMR spectrometry and observed a strong correlation between urine pH, sodium, bicarbonate, alpha-ketoglutarate, citrate, fumarate, glutamate, methylamine, N-methyl-4-pyridone-3-carboxamide, succinate, and taurine and the daily MSG intake. Following the multiple linear regression analysis a simple formula model based on urinary Na^+^, citrate, and glutamate was most accurate and could be validated for estimating daily MSG intake. In conclusion, we propose that the daily MSG intake correlates with urinary metabolites in a rat model and that this new tool for monitoring the impact of MSG on health measures.

## Introduction

Monosodium glutamate (MSG) is a widely used flavor enhancer, particularly in Asian cuisine and processed foods, known for its umami taste [1]. The acceptable daily intake of MSG is unspecified and it is registered in the category of “Generally recognized as safe” by the US Food and Drug Administration [2]. Besides its beneficial palatability effects, MSG has been applied for nutritional care of the elderly people for improving their quality of life [3] and salt reduction [4]. However, harmful effect of MSG consumption was also reported initially for the Chinese restaurant syndrome, asthma exacerbation, and rhinitis [5]. While double-blind controlled trials have disproven these associations, susceptible individuals may still constitute a fraction of the general population [6–9]. Physiological complications related to MSG toxicity such as hypertension, obesity, and gastrointestinal issues, has been highlighted for the public awareness, where the impact of MSG varies based on its dosage, the method of administration, and the duration of exposure [10]. Several studies have shown a link between higher MSG intake and high body mass index (BMI), as well as overweight conditions, which are risk factors for metabolic syndrome (MetS) and the related chronic kidney disease (CKD) [11]. Evidence from China [12, 13] and Thailand [14] supports this association while opposite results have been reported [15, 16]. These conflicting results imply that the relationship between MSG intake and overweight is inconsistent, and may be due also to the challenges in accurately assessing MSG intake and variations among countries, populations, and settings [17]. For these reasons, a reliable tool is needed to accurately estimate dietary MSG intake.

When intaken with the diet, MSG dissociates into sodium and glutamate, this latter being a major nonessential amino acid involved in protein metabolism, energy production for gut cells, and neurotransmission [18]. In murine models, MSG consumption results in more alkaline urine and higher levels of urinary sodium and bicarbonate, indicating its role as an alkalinizing agent [19]. Specific urinary metabolites can serve as biomarkers for MSG intake [19], suggesting a method for monitoring consumption. Excessive MSG intake has been linked to MetS [14] through altered glutamate metabolism, involving processes like gluconeogenesis, energy production, insulin secretion, and fatty acid synthesis [20]. Hence, urinary metabolites might be a useful tool for assessment daily MSG intake, which might have impact for controlling MetS-related disease.

A predictive formula based on urine metabolites would be an important method for approximating the intake of dietary components, such as the formulas used to estimate dietary sodium intake [21, 22]. Urinary chemicals carry rich evidence of nutritional exposures in an individual, and once a detailed urinary metabolite signature and biomarkers are identified and measured, they can be applied to construct predictive models [23]. Identifying those markers can be done using high throughput metabolomic techniques such as Nuclear Magnetic Resonance (NMR) spectroscopy, Liquid or Gas Chromatography-Mass Spectrometry (LC/GC-MS), and High-Performance Liquid Chromatography (HPLC). Moreover, a previously reported that MSG intake induces alterations in the urine metabolites citrate, α-ketoglutarate, glutamate, malonate, methylamine, dimethylamine, and taurine using ^1^H NMR spectrometry [19].

In this study, we aimed to investigate the urinary metabolic profile related to MSG consumption and develop a predictive formula for estimating daily MSG intake in a rat model. This preclinical study may provide valuable information for monitoring MSG intake in humans and aid in the early prevention and control of MSG-induced MetS-related diseases.

## Materials and methods

### Chemicals

Pure food-grade (99%) Monosodium glutamate and analytical grade chemicals including 3-(Trimethylsilyl)propionic acid-D_4_ sodium salt (TSP) (Santa Cruz Biotechnology, USA), sodium azide (NaN_3_) (European Chemicals Agency, Finland), potassium dihydrogen phosphate (KH_2_PO_4_), and deuterium oxide (D_2_O) (Merck, Switzerland) were used in the study.

### Experimental models

**Fig 1:**
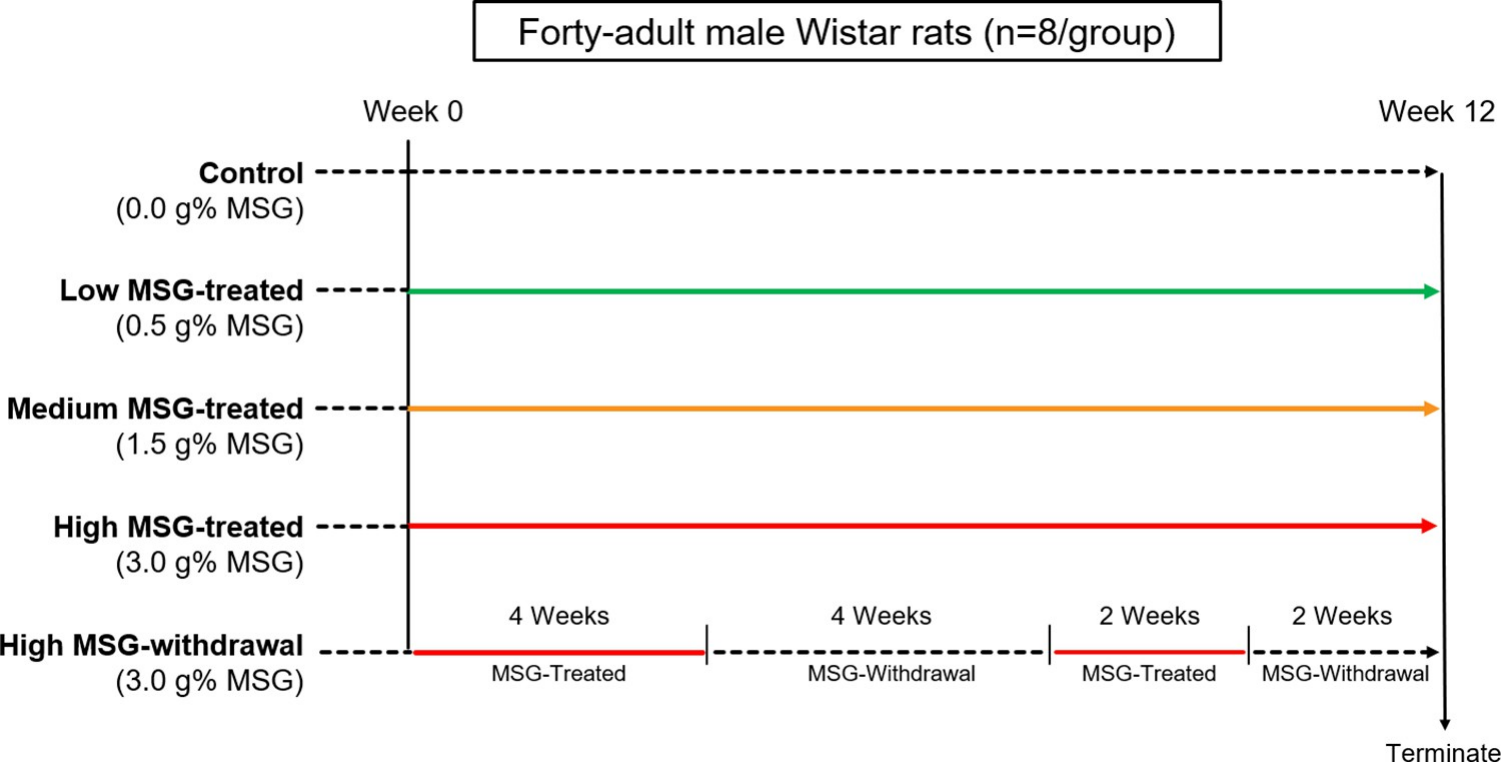
Animal experimental design. Adult male Wistar rats (n = 40) were provided *ad libitum* access to a standard diet and drinking water. Groups 1–4 were designated as MSG-treated groups, with varying doses of MSG administered in drinking water: 0.0 (control), 0.5 (low-MSG-treatment), 1.5 (medium-MSG-treatment), and 3.0 g% (high-MSG-treatment) for 12 weeks. Group 5 was designated as the MSG-withdrawal group, receiving treatment with and without 3.0 g% MSG in drinking water for 4 and 2 consecutive weeks, respectively.

A total of 40 adult male Wistar rats [24], aged 13 weeks, were obtained from the Northeast Laboratory Animal Center (NELAC), Khon Kaen University, Thailand. Animals were acclimatized for 2 weeks before starting the experiment. All animals were housed individually with *ad libitum* access to drinking water and food (Perfect Companion Group, Thailand). The rats were kept in stainless steel cages in a temperature- and humidity-controlled room maintaining a 12-hour light-dark cycle. All the protocols for this study were approved by the guidelines of the Institutional Animal Care and Use Committee of Khon Kaen University under the Ethics of Animal Experimentation of the National Research Council of Thailand (IACUC-KKU-6/63). A total of 40 rats were randomly allocated into five groups with an equal number of animals (N = 8/group). The doses of MSG used in this study were based on our previous study [19] that treated animals with 1% MSG for 2 weeks. In this study, we aimed to explore further by treating the animals as follows: (1) a control group with normal drinking water, (2) a low MSG group receiving 0.5% MSG in drinking water, (3) a medium MSG group receiving 1.5% MSG in drinking water, (4) a high MSG group receiving 3.0% MSG in drinking water for 12 weeks, and (5) an MSG-withdrawal group initially exposed to 3.0% MSG for 4 weeks, followed by 4 weeks of normal drinking water, with this cycle repeated for 2 weeks, as illustrated in Fig 1. The preparation of the MSG drinking water was conducted on a weekly basis. The body weights of the animals were recorded every week, while their food and water intake were carefully recorded on a daily basis. Every two weeks, rats were placed individually in metabolic cages to collect 24-hour urine samples. Urine volume and pH were measured on the same day as sample collection, and all urine samples were stored at -20°C until analysis.

### 24-hour urine collection

Prior collection, the metabolic cages were cleaned thoroughly without the contamination from washing-up reagents, followed by through rinsing with distilled water and air dry. On the collection day, a 9–10 am, the animals were placed into the cage provided with animal food and either MSG-free or MSG-supplemented drinking water for 24 hours. The following day, at the same time, the urine was transferred into 15-ml falcon tubes and recorded the volume. The urine was aliquoted into at least 3 aliquots of 1 ml in 1.5-ml microcentrifuge tubes and stored at -20°C for further analysis.

### Urine pH and electrolytes

Urine pH was measured using a pH meter (Beckman Coulter, USA) standardized against buffer. Urine electrolytes including sodium, potassium, chloride, and bicarbonate were analyzed at the laboratory services in the Faculty of Associated Medical Science, Khon Kaen University, using a quantitative ion-selective electrode-based automatic instrument (Cobas 8000 modular analyzer series, Roche, Switzerland) under standard protocols.

### Urinary glutamate excretion

Urine glutamate was measured by Fluorometric Glutamate Assay Kit #AB138883 (Abcam, United Kingdom), according to the manufacturer’s instructions. Glutamate excretions were calculated by multiplying the concentration with urine volume (milliliter per day).

### Urinary metabolic profile analysis using ^1^H NMR spectrometry

Regarding the availabilities of equipment, materials, and time, urine sample preparation of MSG-treated groups was assigned differ from that of withdrawal group. For MSG-treated groups, urine samples were prepared following established protocols [25], and performed at Vidyasirimedhi Institute of Science and Technology (VISTEC), Rayong, Thailand. Briefly, urine samples were centrifuged to remove particulate matter, and 100 μL of the supernatant was mixed with 300 μL of D_2_O, along with 250 μL of 0.2 M sodium phosphate buffer containing 0.1% TSP, 100% D_2_O, and 3 mM NaN_3_. The mixture was vortexed briefly, and 600 μL of the resulting solution was transferred to 5 mm NMR glass tubes (DWK Life Sciences, Germany) for subsequent NMR analysis. These samples were stored at 4°C until analysis. All ^1^H NMR spectra were acquired at 298.15 K using a Bruker AVIII-600 NMR spectrometer equipped with an inverse cryoprobe, operating at a proton NMR frequency of 600.13 MHz. Each sample underwent 32 scans, with parameters including a spectral width (SW) of 12019.23 Hz, a pulse width (PW) of 10 μs, and a relaxation delay of 1.0 s. The spectra were referenced to a TSP standard solution, ensuring uniform peak height at 0 ppm across all spectra, thus serving as an internal chemical shift standard.

In the withdrawal groups, urine samples were prepared and analyzed in the Department of Nutrition, at the University of California, Davis, USA. Samples were similarly prepared by centrifugation to remove particulate matter, and proteins in the supernatant were removed by centrifugation at 14,000 X g for 30 min through Amicon Ultra 3 kDa spin-filters (Millipore). Additionally, 65 μL of an internal standard (Chenomx Inc., Edmonton, Canada), comprising approximately 5 mM DSS (sodium 2,2-dimethyl-2-silapentane-5-sulfonate) and 0.2% sodium azide in 99% D_2_O, was added to 585 μL of supernatant, as outlined in Slupsky et al [26]. The pH was adjusted to 6.8 ± 0.1 using NaOH or HCl. A 600 μL aliquot of the prepared sample was then transferred to a 5 mm NMR tube (Wilmad Glass Co., New Jersy, United States) and stored at 4°C until NMR data acquisition. All one-dimensional proton NMR spectra of urine samples were acquired using a Bruker AVANCE 600 MHz NMR spectrometer equipped with a SampleJet autosampler, employing the NOESY-presaturation pulse sequence (noesypr). Spectra were acquired at 25°C, with water saturation for 2.5 s during the pre-scan delay, a mixing time of 100 ms, 12 ppm sweep width, an acquisition time of 2.5 s, 8 dummy scans, and 32 transients. Subsequently, all spectra from both groups were zero-filled to 128 K datum points and Fourier transformed with a 0.5-Hz line broadening applied. Manual phasing and baseline correction were performed, followed by metabolite identification and quantification using NMR Suite v9.0 (Chenomx Inc., Edmonton, Canada) [27]. Quantification involved comparing the integral of a known reference signal with signals derived from a library of compounds containing chemical shifts and peak multiplicities. Metabolites were quantified by matching to the Chenomx 600 MHz Library. To account for urine dilution effects, metabolite concentrations were normalized by urine creatinine levels.

### Statistical analysis

Metabolite concentrations are expressed as mean ± standard deviation (SD) and the differences between control, low-, medium-, and high-MSG treated groups were compared for statistical significance using one-way ANOVA. The comparison between with and without MSG-treatment in the withdrawal group was calculated using a paired t-test. Significance was considered when *p* < 0.05. The analyses were performed using GraphPad Prism version 9 (GraphPad Software Inc., USA). To investigate the link between urinary parameters and daily MSG intake, multiple linear regression analyses were conducted in IBM SPSS software version 26 (SPSS Inc., USA). Urinary parameters were treated as independent variables. Model evaluation involved: 1) residual analysis to assess normality and homoscedasticity, 2) examination of R-squared values to measure explained variance, 3) collinearity diagnosis using Variance Inflation Factor (VIF) scores to detect multicollinearity, and 4) Durbin-Watson statistics to identify autocorrelation in residuals. These steps ensure the reliability of the regression model and its interpretations.

## Results

### MSG consumption increases water intake and urine output

**Fig 2:**
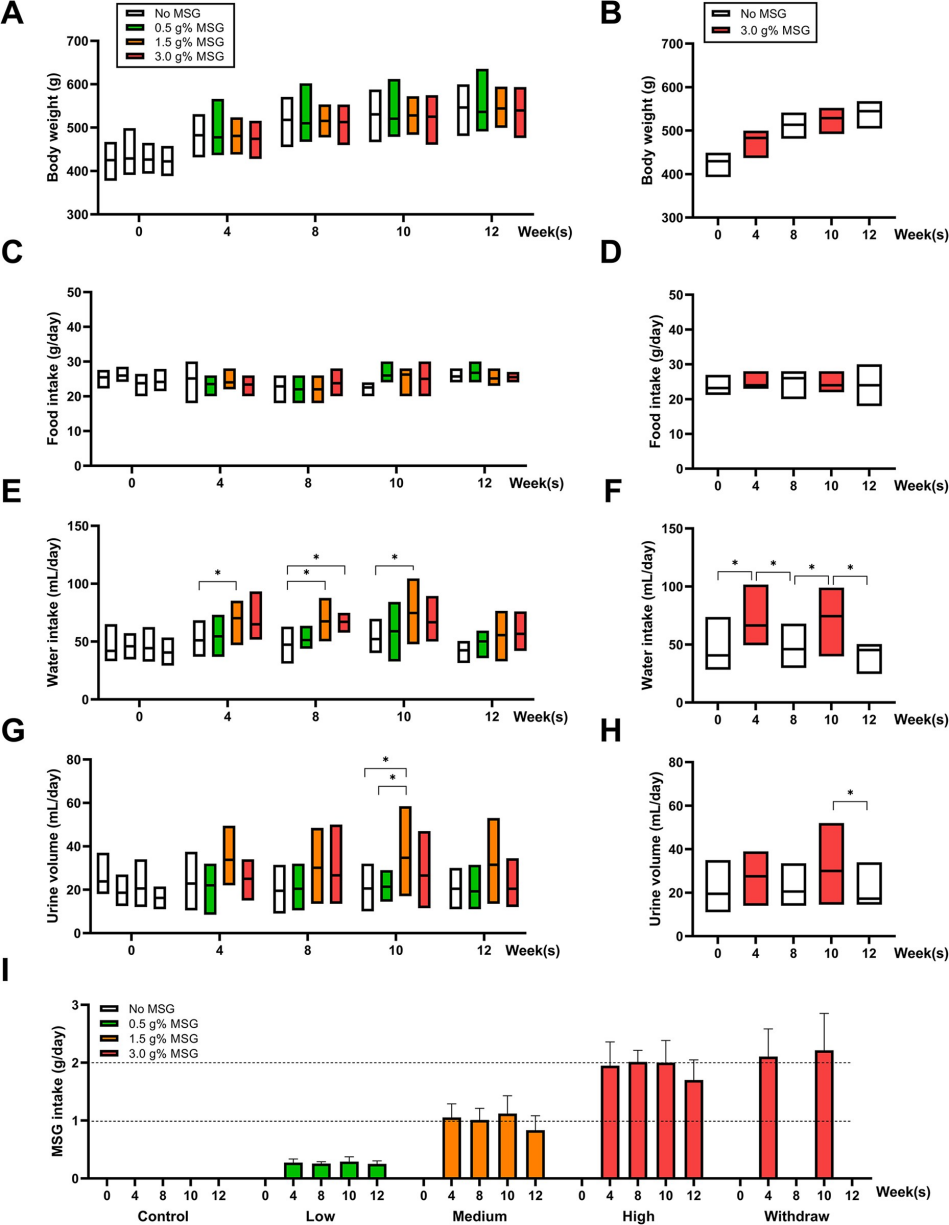
Effects of MSG treated and withdrawal on body weight. (A, B), food intake (C, D), water intake (E, F), and urine output (G, H) during 12 weeks of experiment. Average of MSG intake (I) was monitored at week 0, 4, 8, 10, and 12, based on their water intake. In Fig A-H, data are presented in interleaved low-high plots with mean lines, whereas Fig I is represented as a bar graph showing mean values ± SD. Statistical significance between MSG treated groups (A, C, E, and G) were assessed using one-way ANOVA, and significance within the withdrawal group was determined using the paired t-test (*p* < 0.05).

No significant differences were observed in the body weight changes among animals treated with varying doses of MSG at any time point (Fig 2A and 2B). Similarly, there were no significant differences detected in food intake among the different dosage groups (Fig 2C and 2D). Notably, water intake increased in animals treated with medium and high doses of MSG, particularly in the medium dose group (Fig 2E). The effects of MSG significantly influencing water intake was confirmed in withdrawal group (Fig 2F). Similarly, the urine volume increased in medium MSG group (Fig 2G) and was confirmed in withdrawal group (Fig 2H). Since MSG was added in the drinking water and animals had free access to water, the MSG intake (g/day) was calculated individually based on their water consumption as depicted in Fig 2I. The low MSG-treated group, receiving drinking water mixed with 0.5 g% MSG, had an average MSG intake of 0.27 ± 0.06 g/day. The medium MSG-treated group, receiving water mixed with 1.5 g% MSG, had an average MSG intake of 1.01 ± 0.26 g/day. In the high-MSG treated group and withdrawal group, where drinking water was mixed with 3.0 g% MSG, the animals received an average MSG intake of 1.92 ± 0.35 g/day and 2.16 ± 0.55 g/day, respectively.

### MSG consumption increases urine pH, sodium, and bicarbonate

**Fig 3:**
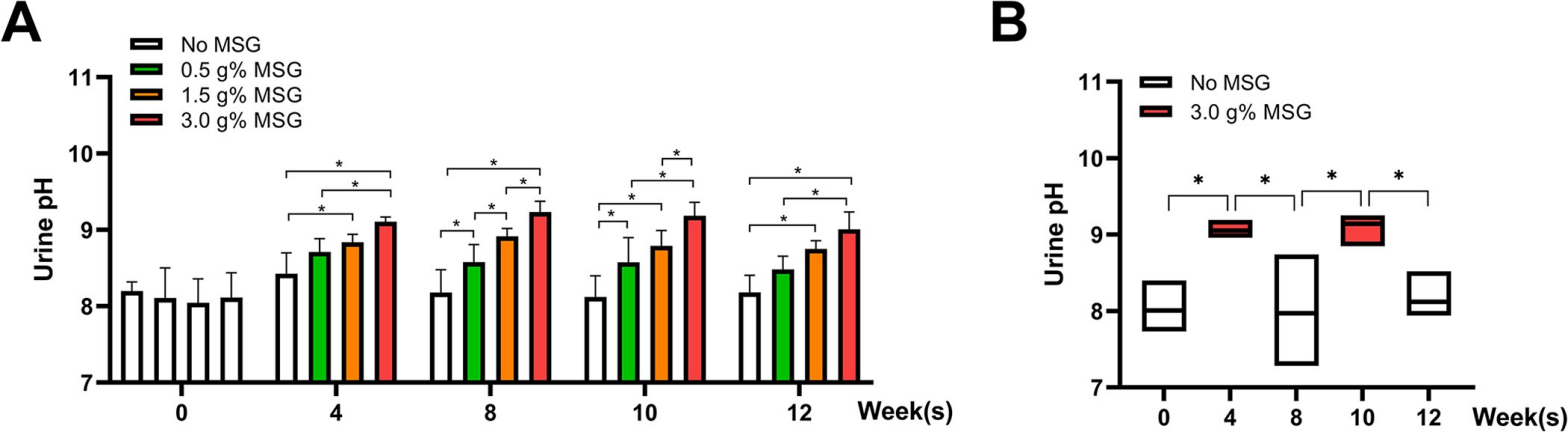
Effects of MSG consumption on urine pH at week 0, 4, 8, 10, 12 of the MSG-treatment group (A) and withdrawal group (B). Statistical significance between groups was evaluated using one-way ANOVA, and significance within withdrawal group was determined using the paired t-test (*p* < 0.05).

**Fig 4:**
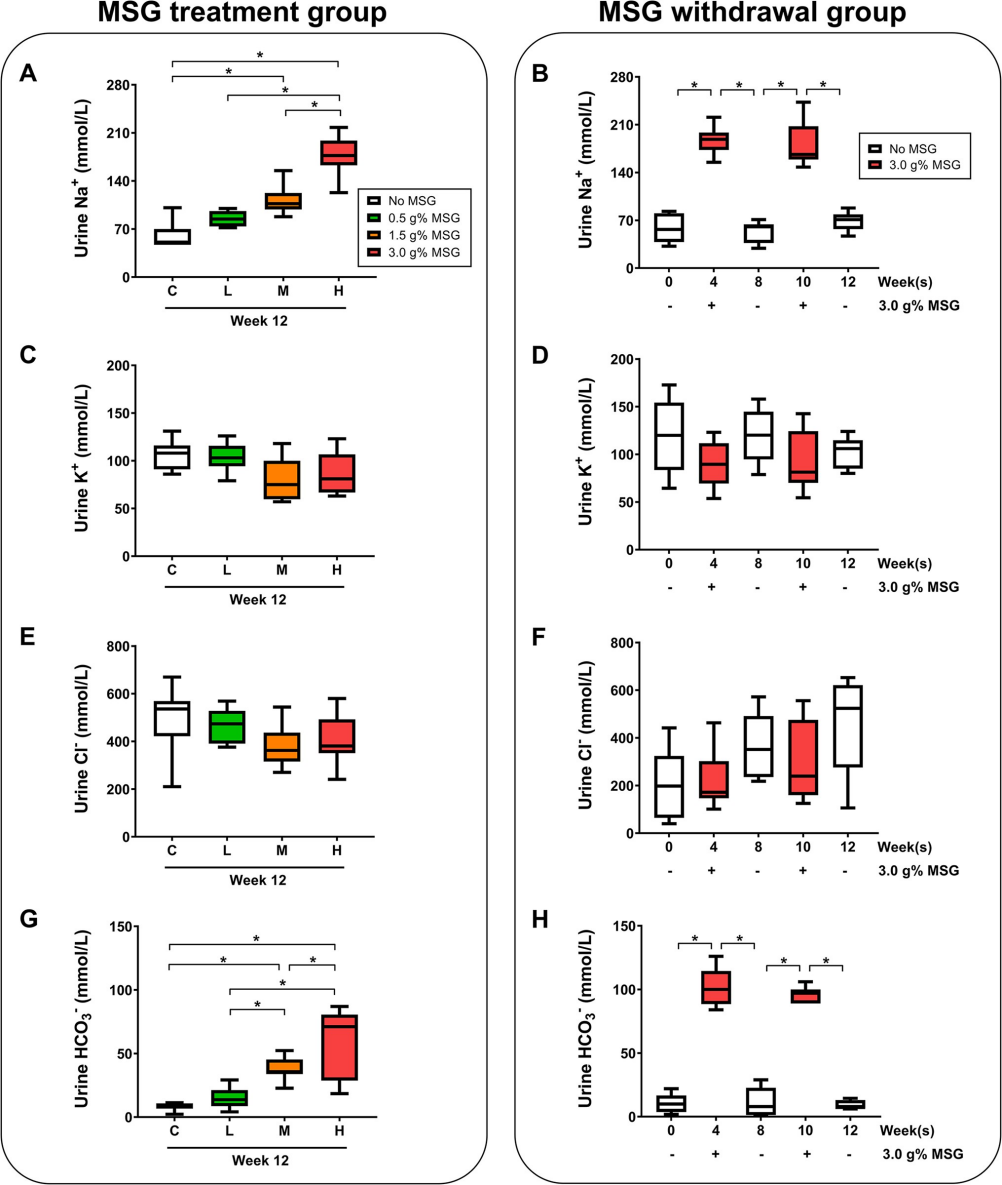
Effects of MSG consumption on urine electrolytes at week 12. Sodium (A), potassium (C), chloride (E), and bicarbonate (G) in MSG-treated groups (C = control, L = low-MSG treated, M = medium MSG-treated, H = high MSG-treated; n = 7-8/group). Additionally, the effects of MSG presence or absence on urine electrolytes at various time points (week 0, 4, 8, 10, and 12) are shown for the MSG-withdrawal group (n = 8) through sodium (B), potassium (D), chloride (F), and bicarbonate (H). Statistical significance between groups was assessed using one-way ANOVA, and significance within group was determined using the paired t-test (*p* < 0.05).

We observed a dose-dependent effect of MSG consumption on urine pH (Fig 3A), which corresponded to the withdrawal treatment, where the urine pH increased during MSG-treatment and dropped back to normal after MSG withdrawal (Fig 3B). A similar phenomenon was observed with urinary electrolytes, sodium, and bicarbonate, which increased with the MSG dose (Fig 4A and 4G), and returned to normal after withdrawal (Fig 4B and 4H). No significant changes were observed in urinary chloride and potassium levels after MSG treatment or withdrawal (Fig 4C–4F).

### MSG consumption increased urinary glutamate excretion

**Fig 5:**
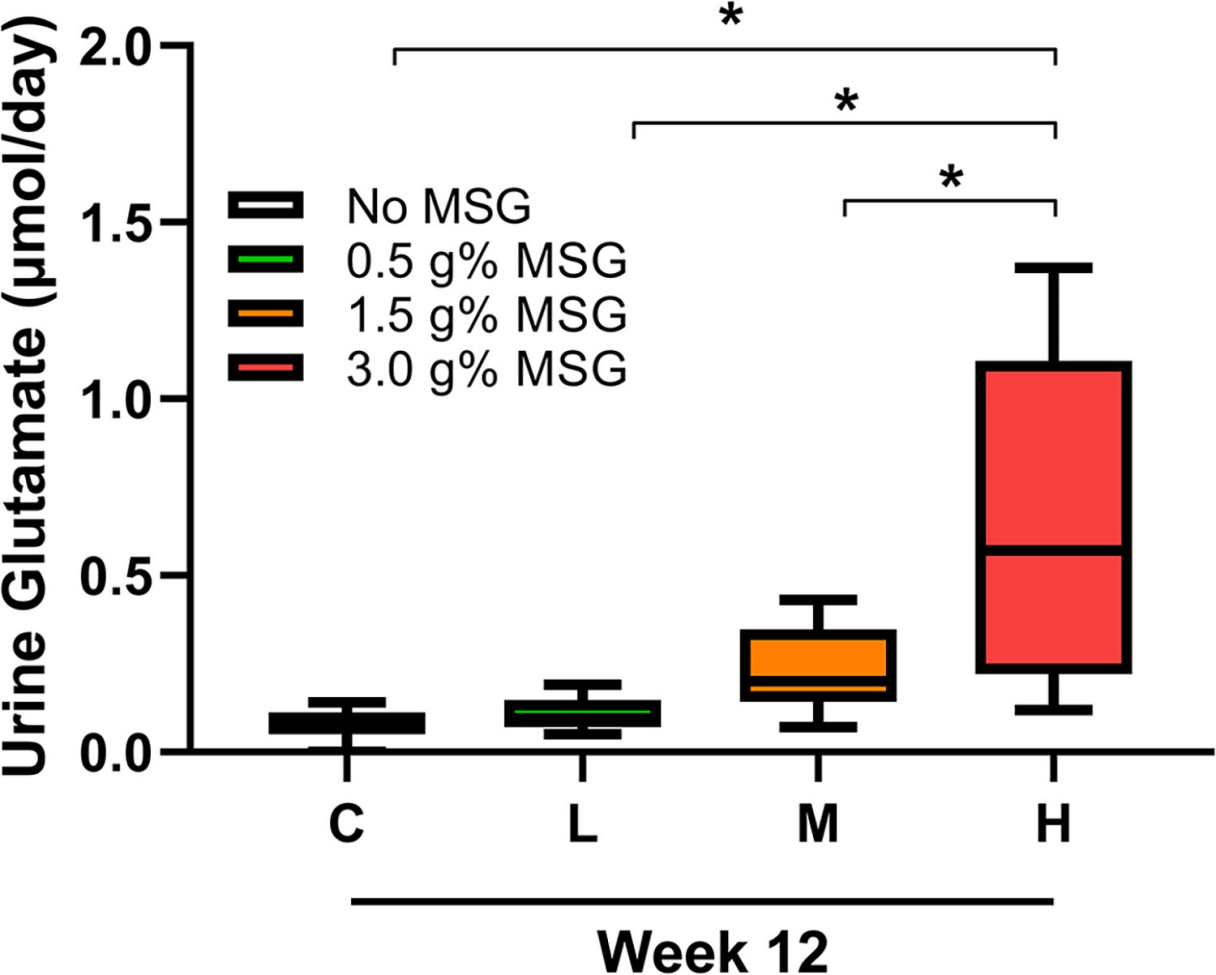
Effects of MSG consumption on urinary glutamate excretion in control (C), low-MSG treated (L), medium-MSG treated (M), and high-MSG treated (H) groups at week 12 of experiment (n = 7-8/group). Data are shown as mean ± SD, the significant different between groups were calculated by one-way ANOVA (*p* < 0.05).

Week-12 urine samples from MSG-treated groups were analyzed to observe the effect of MSG consumption on glutamate renal excretion. The results revealed a significant increase in glutamate excretion only in the high-MSG treated group (Fig 5).

### Quantitation of urinary metabolites

Thirty-two urinary metabolites were detected and found to be shared across both experimental conditions (S1 Table). Sixteen metabolites were observed in urine of animals treated with varying doses of MSG. Eight urinary metabolites, including alpha-ketoglutarate, citrate, dimethylamine, formate, fumarate, glutamate, N,N-dimethylglycine, and succinate, were increased with MSG intake in a dose-dependent manner. Conversely, eight urinary metabolites, namely 2-hydroxyisobutyrate, glycine, methylamine, N-methyl-4-pyridone-3-carboxamide, pantothenate, taurine, trigonelline, and trans-aconitate, were decreased with MSG treatment. In the withdrawal group, fifteen urinary metabolites showed significant differences. Ten urinary metabolites increased during MSG treatment periods, including acetate, alpha-ketoglutarate, betaine, citrate, formate, fumarate, glutamate, N,N-dimethylglycine, pantothenate, and succinate. Additionally, five metabolites decreased with MSG treatment, namely methylamine, N-methyl-2-pyridone-5-carboxamide, N-methyl-4-pyridone-3-carboxamide, N-phenylacetylglycine, and taurine.

**Fig 6:**
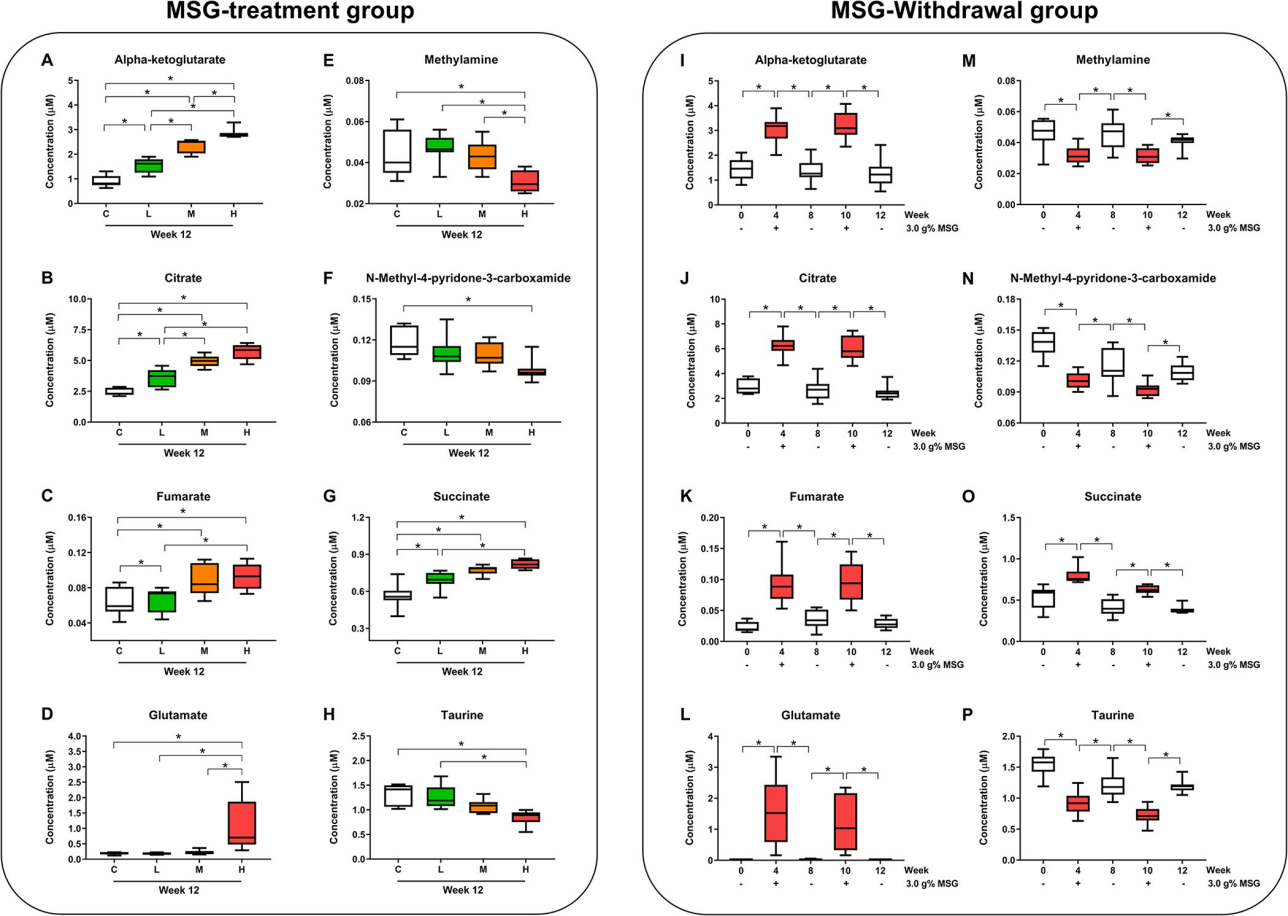
Eight urinary metabolites exhibited significant changes in the MSG-treated groups at week 12, and MSG-withdrawal group at weeks 0, 4, 8, 10, and 12. Five urinary metabolites including alpha-ketoglutarate (A), citrate (B), fumarate (C), glutamate (D), and succinate (G) were increased levels after MSG consumption. In contrast, three metabolites including methylamine (E), N-methyl-4-pyridone-3-carboxamide (F), and taurine (H) showed significant decreased after MSG consumption and elevated after MSG withdrawal. The statistical significance between groups was assessed using one-way ANOVA, and significance within withdrawal group between with and without MSG consumption was determined using the paired t-test (p < 0.05). Groups are labeled as follows: C = control, L = low-MSG treated, M = medium-MSG treated, and H = high-MSG treated groups (n = 7-8/group).

In summary, eight urinary metabolites exhibited significant changes in the MSG-treated groups in a dose-dependent manner (Fig 6A) as well as after MSG-withdrawal (Fig 6B). Among these, five urinary metabolites including alpha-ketoglutarate, citrate, fumarate, glutamate, and succinate demonstrated increased levels after MSG consumption and declined after MSG withdrawal. In contrast, three metabolites including methylamine, N-methyl-4-pyridone-3-carboxamide, and taurine showed significant decreased after MSG consumption and elevated after MSG withdrawal.

### Formulation of predictive indexes of MSG consumption

Several urinary parameters in this study exhibited dose-dependent changes with MSG treatment and showed the corresponding result after MSG withdrawal. These findings suggest their potential as predictors of MSG consumption. Urine pH, electrolytes (sodium and bicarbonate), and 8 significant urinary metabolites (alpha-ketoglutarate, citrate, fumarate, glutamate, methylamine, N-methyl-4-pyridone-3-carboxamide, succinate, and taurine) demonstrated notable associations. To estimate MSG intake, multiple linear regression was employed, using the amount of daily MSG consumption as the dependent variable and incorporating all urine parameters. The analysis met the assumptions required for multiple linear regression, ensuring the validity and reliability of the model. We constructed a predictive model reflecting daily MSG consumption using significant urinary parameters from MSG-treated groups. Evaluation of all predictive models included several key indicators: an R-square value closed to 1, reflecting strong explanatory power; VIF scores below 10, ensuring the absence of multicollinearity and independence of predictors; and Durbin-Watson statistics falling within the ideal range of 1.5–2.5, indicating no autocorrelation. Furthermore, comprehensive model validation was conducted through residual analysis to ensure adherence to normal distributions.

Our findings demonstrate that urine pH alone serves as a potent predictor in a single-factor formula, yielding an R-square value of 0.671 (Table 1). Furthermore, incorporating urine pH alongside urine sodium in a two-factor formula enhances predictive accuracy, yielding an R-square value of 0.738. When using urine metabolites as predictors, they achieved the higher R-square we obtained from 0.685(AKG+TAU), 0.849(CIT+GLU), 0.856(AKG+GLU+TAU), 0.884 (CIT+GLU+TAU), 0.892(AKG+CIT+GLU+TAU), 0.895(CIT+FUM+GLU+TAU) to 0.905 (AKG+CIT+FUM+GLU+TAU). Moreover, combining urine chemistry markers with urinary metabolites yield even higher predictive values of R-square were observed: 0.746(Na+SUC), 0.826(pH+GLU), 0.889(pH+CIT+GLU), 0.897(Na+CIT+GLU), 0.915 (pH+HCO+CIT+GLU), 0.927(Na+HCO+CIT+GLU), 0.929(Na+HCO+CIT+GLU+SUC) to 0.930(Na+HCO+CIT+GLU+MMA).

**Table 1:** Predictive formula of daily MSG consumption derived from 11 urinary parameters and model evaluations.

Urine parameters	Predictive formula of MSG intake (g/day)		R-square	VIF score (<10)	Durbin-Watson statistics (1.5–2.5)
**Urine chemistries**					
pH	MSG (g/day) = 1.598**(pH)** - 13.053		0.671	1.000	1.602
pH + Na	MSG (g/day) = 0.774**(pH)** + 0.007**(Na)** - 6.745		0.738	3.668	2.146
**Urine metabolites**					
AKG + TAU	MSG (g/day) = 0.428**(AKG)** - 1.324**(TAU)** + 1.409		0.685	1.116	1.759
CIT + GLU	MSG (g/day) = 0.312**(CIT)** + 0.564**(GLU)** - 0.834		0.849	1.350	1.748
AKG + GLU + TAU	MSG (g/day) = 0.272**(AKG)** + 0.592**(GLU)** - 1.028**(TAU)** + 1.113		0.856	1.363	1.863
CIT + GLU + TAU	MSG (g/day) = 0.231**(CIT)** + 0.560**(GLU)** - 0.624**(TAU)** + 0.201		0.884	1.843	1.616
AKG + CIT + GLU + TAU	MSG (g/day) = 0.109**(AKG)** + 0.178**(CIT)** + 0.539**(GLU)** - 0.691**(TAU)** + 0.309		0.892	3.341	1.702
CIT + FUM + GLU + TAU	MSG (g/day) = 0.137**(CIT)** + 6.686**(FUM)** + 0.569**(GLU)** - 0.606**(TAU)** + 0.150		0.895	5.030	1.834
AKG + CIT + FUM + GLU + TAU	MSG (g/day) = 0.127**(AKG)** + 0.064**(CIT)** + 7.454**(FUM)** + 0.546**(GLU)** - 0.682**(TAU)** + 0.270		0.905	7.129	1.939
**Combined parameters**					
Na + SUC	MSG (g/day) = 0.010**(Na)** + 1.392**(SUC)** - 1.367		0.746	2.029	1.788
pH + GLU	MSG (g/day) = 1.141**(pH)** + 0.579**(GLU)** - 9.360		0.826	1.354	1.751
pH + CIT + GLU	MSG (g/day) = 0.619**(pH)** + 0.203**(CIT)** + 0.496**(GLU)** - 5.684		0.889	2.482	1.878
Na + CIT + GLU	MSG (g/day) = 0.005**(Na)** + 0.193**(CIT)** + 0.486**(GLU)** - 0.854		0.897	2.508	2.111
pH + HCO + CIT + GLU	MSG (g/day) = 0.153**(pH)** + 0.010**(HCO)** + 0.208**(CIT)** + 0.327**(GLU)** - 1.925		0.915	4.991	1.684
Na + HCO + CIT + GLU	MSG (g/day) = 0.003**(Na)** + 0.009**(HCO)** + 0.174**(CIT)** + 0.323**(GLU)** - 0.748		0.927	3.335	1.892
Na + HCO + CIT + GLU + SUC	MSG (g/day) = 0.003**(Na)** + 0.008**(HCO)** + 0.163**(CIT)** + 0.328**(GLU)** + 0.183**(SUC)** - 0.836		0.929	3.819	1.725
Na + HCO + CIT + GLU + MMA	MSG (g/day) = 0.002**(Na)** + 0.009**(HCO)** + 0.178**(CIT)** + 0.311**(GLU)** - 4.871**(MMA)** - 0.497		0.930	3.762	1.974

Note: The table presents the predictive formula for MSG consumption (g/day), derived from the analysis of 11 concentrations of urinary parameters which in mM for urine chemistry and μM for urine metabolites. The integration of 12-week MSG-treated (0.0, 0.5, 1.5, and 3.0 g% in drinking water) and withdrawal data (collected at weeks 0, 4, 8, 10, and 12) was utilized to develop a precise predictive model. All predictive models underwent rigorous assessment for normal distribution through residual analysis. Additionally, the outcomes of model evaluations, including Coefficient of Determination (R-squared) and assessment of multicollinearity (VIF score), were provided. Furthermore, the evaluation of linear regression models, particularly in the context of autocorrelation, was conducted using Durbin-Watson statistics. Abrevation; HCO = bicarbonate, SUC = succinate, AKG = alphaketoglutarate, GLU = glutamate, TAU = taurine, CIT = citrate, FUM = fumarate, MMA = methylamine

### The predictive model validation

**Fig 7:**
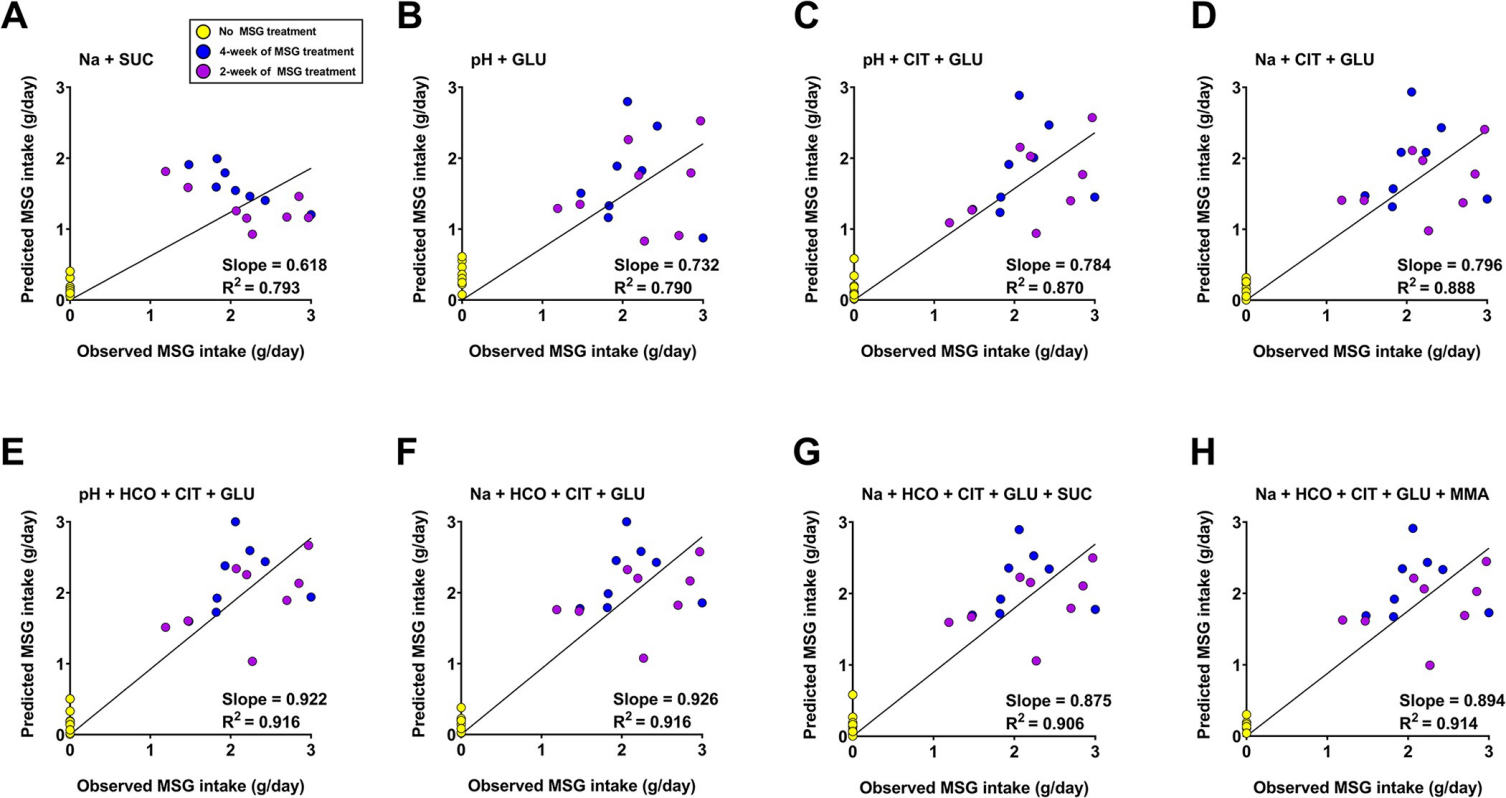
Model validation using data from MSG-withdrawal group. The combined model were validated using concentration of urine chemistry (mM) and metabolites (μM) in withdrawal groups in week 0, 4, 8, 10, and 12. The symbol was describe as following: Green circle refer to the no MSG treatment period (Week 0, 8, and 12), Blue circle refer to the 4-week of 3.0 g% MSG treatment period (week 4), and violet circle refer to the 2-week of 3.0 g% MSG treatment period (week 12).

The predictive model reported here underwent validation using urinary data from a withdrawal group (Week 0, 4, 8, 10, and 12). This validation process involved analyzing four different datasets from the withdrawal group: 1) Baseline level (No MSG treatment), 2) Week 4 (After 4 weeks of 3.0 g% MSG treatment), 3) Week 8 (After 2 weeks of 3.0 g% MSG treatment), and 4) Withdrawal periods in both after 4-week and 2-week wash out periods. To predict daily MSG intake, various combined equations ranging from two to five components, were employed to predict MSG concentration, resulting in high correlation coefficients (R-square > 0.79), as depicted in Fig 7.

## Discussion

Assessment urinary metabolites could reflect exposure agents, such as radiation exposure [23]. In this study, we investigated the urinary metabolic profile proportional to MSG consumption and create the predictive formula for the estimate of the daily MSG intake in an established animal model previously used in our MSG work. Among the 24-hour urinary metabolites and chemistries, three parameters of urine chemistries and eight urinary metabolites had positive association with MSG intake and ultimately a combination of sodium, citrate, and glutamate could best estimate MSG intake.

Adult male Wistar rats were randomly assigned into groups providing MSG at varying concentrations in drinking water (0, 0.5, 1.5, and 3.0 g%) for 12 weeks and arbitrary classified as dose-response of no, low-, medium-, and high- MSG consumption, in which animals received MSG intake of 0, 0.27 ± 0.06, 1.01 ± 0.26, 1.92 ± 0.35 g/day, respectively. This dosage is equivalent to 0, 8, 15, and 35 g/day, respectively, MSG intake for a 60-kg person [28]. Regarding the MSG dosage, there have been reports of 8–12 g in single and double-blind studies [7, 29], and 14 g/day in a cross-sectional study [14]. Moreover, a tablespoon (15 g) MSG or even a half rice ladle spoon (30 g) added in a meal such as papaya salad can be observed in a daily life in Thailand, therefore, the MSG doses applied to animal in this present study is realistic to the consumption observed in several Countries, especially in the East.

The strong correlation between urine pH and urine electrolytes (sodium, bicarbonate) with daily MSG intake in a dose-dependent manner observed in this study appears as robust. MSG consumption increased urinary pH has been reported previously in rats receiving 1% MSG in drinking water for 2 weeks [19], 20% dietary MSG for 9 weeks [30], 6% dietary MSG for 3 months [31], or 2 mg/g body weight MSG/day in drinking water for 9 months [32]. An increase of urinary pH is a common feature associated to MSG consumption in rodents, independent of the diet or water intake and this study confirms the changes as a dose-dependent manner and can be reversible, as illustrated by the MSG withdrawal group (Fig 3) which suggests a reversible effect of a temporary MSG intake.

The urinary pH changes correspond to the urinary bicarbonate changes. Since the kidney plays a major role in the homeostasis of plasma and urine pH by acid excretion and bicarbonate reabsorption via the ion exchanger channels [33], the increase of bicarbonate level in urine indicating its excess in plasma and less reabsorption in the renal tubular system. This was also observed in our previous study that rats which received 1 g% MSG in drinking water had higher urine bicarbonate levels as well as the lower expression of three major ion exchangers, carbonic anhydrase 2 *(CAII)*, Na^+^-HCO_3_^-^ co-transporter 1 *(NBC-1)*, and anion exchanger 1 *(AE1)* in kidney cortex compared with controls [19]. The low expression *NBC-1* was previously illustrated in rats that received bicarbonate loading [34]. Since the bicarbonate in the body derived from cellular respiration [35], MSG consumption may increase glutamate as the substrate of citric acid cycle where the CO_2_ is generated, released to the blood circulation, and converted into bicarbonate under carbonic anhydrase activity [33]. It has been reported that 95% of fed glutamate was metabolized by intestinal epithelium and of this 50% is oxidized to CO_2_ [18] and this serves as a site for food digestion, nutrient absorption, and metabolism, the gastrointestinal tract becomes the major organ of bicarbonate generation second only to the liver [36, 37]. The excess of bicarbonate is then transported to the kidneys and filtered out through glomerulus, tubular lumen, and bladder creating alkaline urine. Thus, the higher MSG consumption, the greater the urinary bicarbonate was observed in MSG-treated groups (Fig 4). Moreover, the finding of urinary metabolites such as glutamate, alpha-ketoglutarate, succinate, fumarate, and citrate increased with the dose of MSG applied (Fig 6) corresponding with our previous study [19]. The mechanism by which MSG increased levels of intermediates of tricarboxylic acid (TCA) cycle in urine is in agreement with how these organic anions are handled and excreted by the kidneys which was clearly demonstrated by Packer and team [38]. Therefore, the urinary metabolic profile found in this present study is mainly from consequence of alkaline urine.

Using the multiple linear regression model, we obtained several predictive formulas (Table 1), generating from eleven urinary parameters that related to the MSG intake. Quite surprisingly, only the urine chemistry parameters, either as a single (urine pH) or combination (urine pH and Na) were a good predictor for MSG intake. This suggested the feasibility of testing our hypothesis in the research field where the high-throughput machines and techniques such as HPLC, LC/MS, or NMR are not available. With the full function of equipment and facilities, formulas from urine chemistries and urinary metabolites can be obtained with higher precision on MSG estimates. However, in terms of translation, the formulas with bicarbonate and methylamine parameters may be hampered, because bicarbonate level is very low in human urine and methylamine is derived from choline metabolism via the gut microbiota [39] which may create the difference among species.

**Fig 8:**
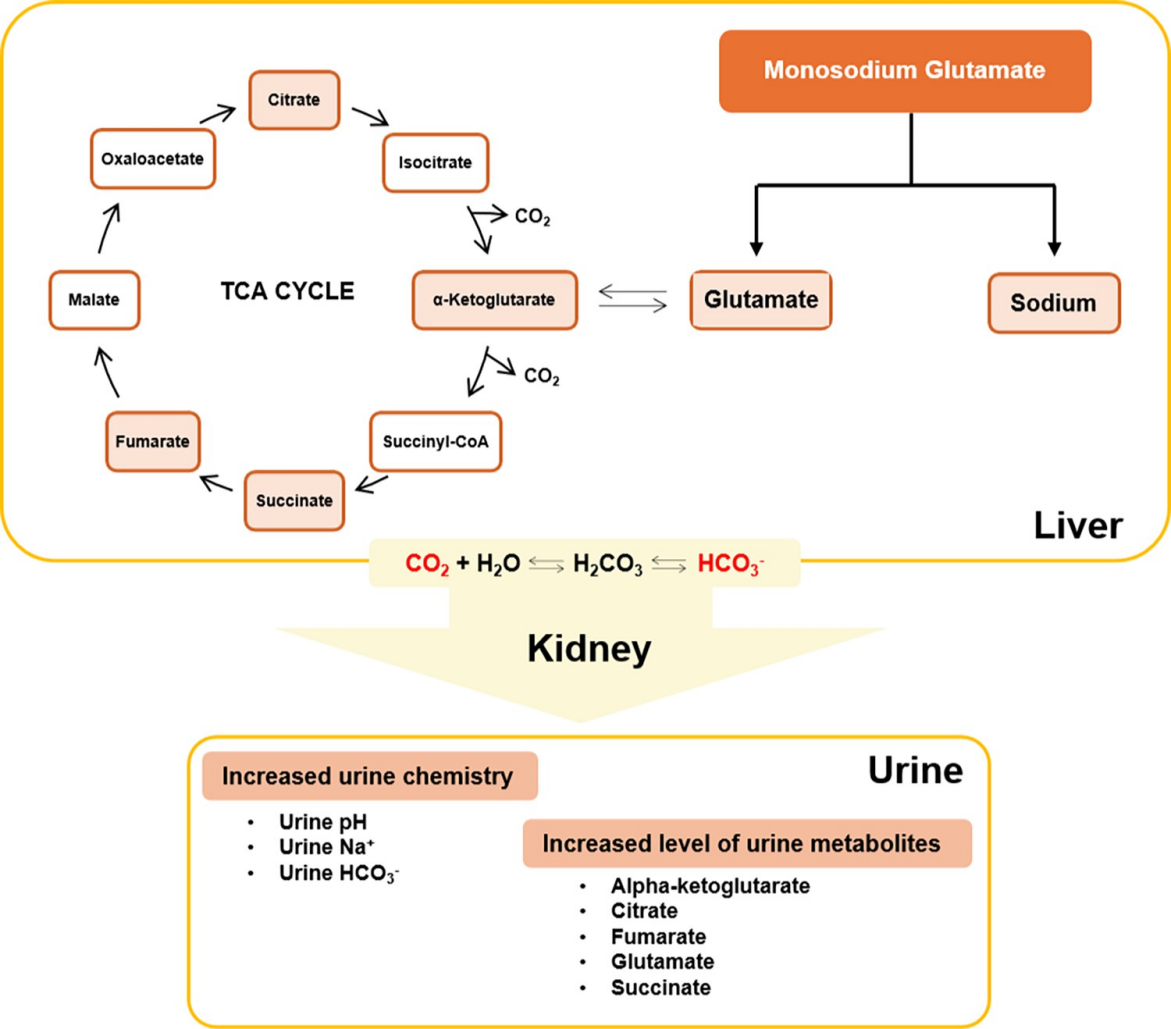
In our proposed model, after MSG consumption, sodium ions and glutamate are absorbed in the intestinal villi, transported to the liver and other organs via the bloodstream, and excreted through urine when in excess. As a consequence, urinary Na^+^ levels are observed to increase in a dose-dependent manner. Glutamate derived from dietary protein and MSG is utilized by the intestinal epithelium, with excess absorbed and distributed to the liver and other organs for via blood circulation. In the catabolic pathway, glutamate enters the tricarboxylic acid cycle (TCA cycle) through alpha-ketoglutarate by transamination, undergoing oxidation to produce intermediates of the citric acid cycle and carbon dioxide (CO_2_) as the final product. After MSG consumption, the excess bicarbonate resulting from CO_2_ is excreted in urine, leading to alkaline urine in dose-dependent manner. Elevated urinary pH impacts the reabsorption of metabolites, particularly divalent anion substances, potentially explaining the presence of alpha-ketoglutarate, citrate, fumarate, succinate, and glutamate in the urine of animals following MSG consumption in a dose-dependent manner.


Taken together, we prefer the simple formula combining Na^+^, citrate, and glutamate together because it represents the MSG consumption as its inherent structure and metabolism supported by the high score of accuracy and validation (Fig 7D). How the Na^+^, citrate, and glutamate related to the MSG consumption is summarized in Fig 8. Glutamate metabolism at least in gut is highly conserved among species, piglets, rat, and human [40, 41]. Therefore, we proposed the equation for estimating daily MSG consumption derived from its metabolites as below, where the concentration of each parameter in urine is converted to micromolar (μM). We believe this tool may provide conclusive data on the health consequences of MSG consumption in future studies.

MSGintake(g/day)=0.005[Na]+0.193[citrate]+0.486[glutamate]‐0.854

We acknowledge that the inclusion only of 13-week-old rats, without younger animals, is a possible limitation that should be addressed in future studies, along with other species.

In conclusion, the present study provides a demonstration of the urinary metabolic changes with dose-dependence after MSG consumption in animal model. We demonstrated that MSG consumption increases urinary pH, sodium, and bicarbonate, leading to alteration of urinary metabolic profiles in a dose-dependent manner and can be exploited as a predictive formula of its consumption in animal model. We, propose herein a novel formula to estimate the daily MSG intake based on the urinary MSG metabolites. This may be used as a relevant tool for estimating MSG consumption in determining MetS-related diseases such as chronic kidney disease in future studies.

## Supporting information

S1 Table Urinary metabolite concentrations of MSG-treated groups (Week 12) and MSG-withdrawal group (Week 0, 4, 8, 10, and 12). (DOCX)
