# Supplementary material for: Urinary metabolic profile and its predictive indexes after MSG consumption in rat
Source: PLoS One. 2024 Sep 3;19(9):e0309728. doi: 10.1371/journal.pone.0309728 (PMC11371250; doi:10.1371/journal.pone.0309728)
Supplement: S1 Table — (DOCX) [file pone.0309728.s001.docx]

**S1 Table.** **Urinary metabolite concentrations** **of MSG-treated groups (Week 12) and MSG-withdrawal group (Week 0, 4, 8, 10, and 12).**

| **METABOLITES** | **MSG dose-dependent groups** | | | | | **MSG withdrawal group** | | | | | | | | |
| --- | --- | --- | --- | --- | --- | --- | --- | --- | --- | --- | --- | --- | --- | --- |
|  | **Concentration (µM)** | | | | ***p*-value** | **Concentration (µM)** | | | | | ***p*-value** | | | |
|  | **Control** | **Low** | **Medium** | **High** |  | **Week 0** | **Week 4** | **Week 8** | **Week 10** | **Week 12** | **0 vs 4** | **4 vs 8** | **8 vs 10** | **10 vs 12** |
| 1-Methylnicotinamide | 0.011 | 0.011 | 0.009 | 0.011 | 0.862 | 0.020 | 0.014 | 0.019 | 0.013 | 0.016 | 0.021 | 0.114 | 0.099 | 0.044 |
| 2-Hydroxyisobutyrate | 0.048 | 0.051 | 0.048 | 0.042 | 0.007 | 0.067 | 0.049 | 0.043 | 0.040 | 0.046 | 0.004 | 0.054 | 0.170 | 0.003 |
| 3-Indoxylsulfate | 0.115 | 0.126 | 0.122 | 0.128 | 0.418 | 0.114 | 0.127 | 0.122 | 0.123 | 0.096 | 0.103 | 0.563 | 0.909 | 0.001 |
| Acetate | 0.105 | 0.169 | 0.141 | 0.151 | 0.231 | 0.137 | 0.161 | 0.082 | 0.170 | 0.102 | 0.014 | <0.001 | 0.010 | 0.028 |
| Alanine | 0.029 | 0.031 | 0.027 | 0.025 | 0.120 | 0.031 | 0.027 | 0.026 | 0.032 | 0.024 | 0.050 | 0.369 | <0.001 | 0.001 |
| **Alpha-ketoglutarate** | **0.916** | **1.539** | **2.340** | **2.495** | **<0.001** | **1.430** | **3.056** | **1.378** | **3.211** | **1.287** | **<0.001** | **<0.001** | **<0.001** | **<0.001** |
| Betaine | 0.066 | 0.077 | 0.082 | 0.088 | 0.134 | 0.083 | 0.104 | 0.060 | 0.077 | 0.059 | 0.006 | <0.001 | <0.001 | <0.001 |
| Choline | 0.027 | 0.033 | 0.023 | 0.023 | 0.062 | 0.028 | 0.028 | 0.024 | 0.024 | 0.023 | 0.692 | 0.100 | 0.974 | 0.642 |
| **Citrate** | **2.404** | **3.630** | **4.953** | **5.672** | **<0.001** | **2.971** | **6.249** | **2.748** | **6.020** | **2.481** | **<0.001** | **<0.001** | **<0.001** | **<0.001** |
| Dimethylamine | 0.047 | 0.097 | 0.100 | 0.088 | <0.001 | 0.100 | 0.083 | 0.085 | 0.077 | 0.070 | 0.110 | 0.777 | 0.417 | 0.475 |
| Formate | 0.062 | 0.067 | 0.097 | 0.093 | <0.001 | 0.086 | 0.104 | 0.058 | 0.082 | 0.049 | 0.269 | <0.001 | 0.006 | <0.001 |
| **Fumarate** | **0.035** | **0.057** | **0.073** | **0.083** | **<0.001** | **0.023** | **0.093** | **0.036** | **0.096** | **0.029** | **<0.001** | **<0.001** | **<0.001** | **<0.001** |
| Glucose | 0.298 | 0.285 | 0.280 | 0.250 | 0.413 | 0.483 | 0.243 | 0.338 | 0.269 | 0.269 | 0.005 | 0.265 | 0.166 | 0.378 |
| **Glutamate** | **0.187** | **0.186** | **0.223** | **1.042** | **<0.001** | **0.000** | **1.479** | **0.007** | **1.091** | **0.000** | **0.013** | **0.014** | **0.019** | **0.017** |
| Glycine | 0.107 | 0.139 | 0.082 | 0.102 | <0.001 | 0.118 | 0.108 | 0.099 | 0.096 | 0.084 | 0.046 | 0.161 | 0.755 | 0.187 |
| Hippurate | 0.687 | 0.720 | 0.709 | 0.656 | 0.175 | 0.846 | 0.744 | 0.678 | 0.641 | 0.640 | 0.057 | 0.041 | 0.320 | 0.908 |
| Isoleucine | 0.005 | 0.006 | 0.005 | 0.005 | 0.680 | 0.006 | 0.005 | 0.005 | 0.006 | 0.006 | 0.593 | 0.903 | 0.318 | 0.587 |
| **Methylamine** | **0.044** | **0.047** | **0.043** | **0.031** | **0.002** | **0.046** | **0.032** | **0.046** | **0.031** | **0.041** | **0.006** | **0.005** | **0.025** | **0.002** |
| N,N-Dimethylglycine | 0.022 | 0.024 | 0.025 | 0.032 | 0.004 | 0.025 | 0.027 | 0.015 | 0.019 | 0.015 | 0.796 | <0.001 | 0.023 | 0.005 |
| N-Isovaleroylglycine | 0.035 | 0.033 | 0.028 | 0.029 | 0.057 | 0.032 | 0.031 | 0.031 | 0.029 | 0.034 | 0.285 | 0.805 | 0.067 | <0.001 |
| N-Methyl-2-pyridone-5-carboxamide | 0.009 | 0.009 | 0.008 | 0.008 | 0.865 | 0.012 | 0.008 | 0.010 | 0.007 | 0.009 | 0.002 | 0.055 | 0.044 | 0.031 |
| **N-Methyl-4-pyridone-3-carboxamide** | **0.118** | **0.110** | **0.109** | **0.098** | **0.005** | **0.137** | **0.101** | **0.114** | **0.093** | **0.110** | **<0.001** | **0.033** | **0.017** | **0.002** |
| N-Phenylacetylglycine | 0.211 | 0.230 | 0.244 | 0.222 | 0.406 | 0.198 | 0.181 | 0.215 | 0.171 | 0.148 | 0.437 | 0.010 | 0.020 | 0.016 |
| Pantothenate | 0.047 | 0.049 | 0.047 | 0.041 | <0.001 | 0.055 | 0.047 | 0.045 | 0.039 | 0.048 | 0.014 | 0.106 | 0.016 | <0.001 |
| **Succinate** | **0.562** | **0.689** | **0.772** | **0.820** | **<0.001** | **0.541** | **0.800** | **0.412** | **0.625** | **0.386** | **0.001** | **<0.001** | **<0.001** | **<0.001** |
| **Taurine** | **1.332** | **1.267** | **1.075** | **0.848** | **<0.001** | **1.543** | **0.918** | **1.216** | **0.722** | **1.200** | **<0.001** | **0.020** | **0.001** | **<0.001** |
| Trigonelline | 0.082 | 0.088 | 0.083 | 0.077 | <0.001 | 0.101 | 0.085 | 0.077 | 0.073 | 0.072 | 0.015 | 0.013 | 0.294 | 0.646 |
| Trimethylamine N-oxide | 0.075 | 0.064 | 0.073 | 0.079 | 0.363 | 0.092 | 0.072 | 0.083 | 0.062 | 0.060 | 0.046 | 0.163 | 0.008 | 0.684 |
| Urea | 105.466 | 117.021 | 113.099 | 110.539 | 0.122 | 105.817 | 117.278 | 101.423 | 92.927 | 78.327 | 0.040 | <0.001 | 0.175 | 0.003 |
| Valine | 0.010 | 0.011 | 0.010 | 0.010 | 0.782 | 0.013 | 0.011 | 0.010 | 0.012 | 0.010 | 0.034 | 0.225 | 0.087 | 0.147 |
| trans-Aconitate | 0.049 | 0.052 | 0.047 | 0.044 | 0.009 | 0.062 | 0.046 | 0.048 | 0.041 | 0.043 | 0.001 | 0.393 | 0.005 | 0.266 |
| β-Pseudouridine | 0.092 | 0.096 | 0.095 | 0.090 | 0.061 | 0.114 | 0.093 | 0.094 | 0.082 | 0.080 | 0.002 | 0.833 | 0.007 | 0.571 |
